# Government use licenses in Thailand: an assessment of the health and economic impacts

**DOI:** 10.1186/1744-8603-7-28

**Published:** 2011-08-14

**Authors:** Inthira Yamabhai, Adun Mohara, Sripen Tantivess, Kakanang Chaisiri, Yot Teerawattananon

**Affiliations:** 1 Health Intervention and Technology Assessment Program (HITAP), Bureau of Health Policy and Strategy, Ministry of Public Health, Thailand

## Abstract

**Background:**

Between 2006 and 2008, Thailand's Ministry of Public Health (MOPH) granted government use licenses for seven patented drugs in order to improve access to these essential treatments. The decision to grant the government use licenses was contentious both within and beyond the country. In particular, concerns were highlighted that the negative consequences might outweigh the expected benefits of the policy. This study conducted assessments of the health and economic implications of these government use licenses.

**Methods:**

The health and health-related economic impacts were quantified in terms of i) Quality Adjusted Life Years (QALYs) gained and ii) increased productivity in US dollars (USD) as a result of the increased access to drugs. The study adopted a five-year timeframe for the assessment, commencing from the time of the grant of the government use licenses. Empirical evidence gathered from national databases was used to assess the changes in volume of exports after US Generalized System of Preferences (GSP) withdrawal and level of foreign direct investment (FDI).

**Results:**

As a result of the granting of the government use licenses, an additional 84,158 patients were estimated to have received access to the seven drugs over five years. Health gains from the use of the seven drugs compared to their best alternative accounted for 12,493 QALYs gained, which translates into quantifiable incremental benefits to society of USD132.4 million. The government use license on efavirenze was found to have the greatest benefit. In respect of the country's economy, the study found that Thailand's overall exports increased overtime, although exports of the three US GSP withdrawal products to the US did decline. There was also found to be no relationship between the government use licenses and the level of foreign investment over the period 2002 to 2008.

**Conclusions:**

The public health benefits of the government use licenses were generally positive. Specifically, the policy helped to increase access to patented drugs, while the impact of the US GSP withdrawal did not adversely affect the overall export status. Because the levels of benefit gained from the government use licenses varied widely between the seven drugs, depending on several factors, this study makes recommendations for the future implementation of the policy in order to maximise benefits.

## Background

The World Trade Organization (WTO) Agreement on Trade-Related Aspects of Intellectual Property (TRIPS) sets out the minimum standards for intellectual property protection, including patents for pharmaceuticals [1]. The TRIPS Agreement has been a source of contention and debate within the health community because of the effects that increased levels of patent protection will have on drug prices and access [2,3]. However, the adoption of the Ministerial Declaration on the TRIPS Agreement and Public Health at the WTO Ministerial Conference in Doha, 2001 (the Doha Declaration) affirmed that the TRIPS Agreement does contain a degree of flexibility permitting governments to consider different options when formulating laws and policies related to patent protection and public health, including the use of compulsory licenses^i ^and other so-called "TRIPS flexibilities" [4].

Between November 2006 and January 2008, the Government of Thailand granted a series of compulsory licenses to allow the import of generic equivalents of seven patented drugs into Thailand. These licenses, hereafter referred to as government use licenses, were granted in accordance with Section 51 of the Thai Patent Act B.E. 2522, which permits the government use of patents in the general public interest, so that "any ministry, bureau or department of the Government" can exercise the rights in any patent "to carry out any service for public consumption".

Thailand justified the government use licenses on the grounds that they were needed to ensure access to essential drugs that had been proven effective and necessary for treating diseases with high prevalence that were increasingly becoming major public health concerns in the country. The imported generic drugs were to be for use under the national health insurance scheme. Since 2002, Thailand has provided universal health coverage for its population of 64 million, with all Thai people now covered under one of three national public health insurance schemes [5]. Coverage under the health insurance schemes entitles patients access to drugs on the National List of Essential Medicines (NLEM).

In 2003, the government undertook a commitment to provide universal access to antiretroviral drugs (ARVs). Although the national health budget was increased, it was insufficient to meet the goal of universal access to ARVs in Thailand. The expansion of the ARV treatment program would require greater financing additional financing of approximately 267 million USD, or 2% of the total UC health budget over a five year period for fiscal years 2007-2011 [6]. In addition, epidemiological evidence indicated a rising trend of cardiovascular diseases and cancer as major causes of deaths in Thailand [7]. The public health insurance system could not ensure sufficient access to the needed treatments, due to the high prices of the drugs, many of which were patented and for which no generic version was available in Thailand [8].

The use of generic equivalents of patented drugs was identified as a sustainable cost-containment measure. However, introduction of these generic equivalents necessitated government use licenses to import them into Thailand. The first license, granted in November 2006 was for the ARV drug, efavirenz (EFV). The second and third licenses were granted in January 2007, for the lopinavir/ritonavir (LPV/r) ARV combination and clopidogrel (an antiplatelet agent used in the treatment of coronary artery disease) [9]. Finally, four licenses were granted in January 2008 for cancer drugs, letrozole, docetaxel, erlotinib, and imatinib (which are used in the treatment of breast and lung cancers, gastrointestinal stromal tumor (GIST) and leukaemia) [10].

The decision to grant the government use licenses caused controversy both within and beyond Thailand. The validity of the licenses was initially questioned, particularly the licenses for drugs to treat heart disease and cancer, due to the widespread misconception that compulsory or government use licenses may only be used to address public health crises in pandemic or emergency situations. Criticism of the licenses also came from the Office of the United States Trade Representative (USTR), which elevated Thailand's ranking from a country on the Watch List (WL) to one on the Priority Watch List (PWL) in the USTR's Special 301 Report. In July 2007, the USTR also withdrew duty-free access to the United States (US) market for three Thai products under the US Generalized System of Preferences (GSP)^ii^. This gave rise to concerns in Thailand that such trade sanctions may outweigh the expected benefits of the government use licenses.

Much has already been written on the legal issues of this case [11-13]; hence, this paper does not intend to cover the same ground. It should however be noted that the legal debate has now largely abated with the matter not being formally pursued in the Thai courts, nor has it been referred to the WTO's TRIPS Council or its dispute settlement system. This paper details the findings of a health and economic impact assessment of the government use licenses. This is with the aim of determining the health and health-related economic impacts arising from the grant of the government use licenses, as well as their effect on the national economy and foreign investment. This will provide evidence for improved decision making in Thailand. The methodologies used to assess and quantify the impacts are described in detail below.

## Methods

### The evaluation of health and health-related economic impacts

In terms of the health and health-related economic impacts, the study sought to estimate the following: (i) increase in the number of patients who would gain access to the seven drugs as a result of the government use licenses; (ii) the health gains in terms of Quality-Adjusted Life Years (QALYs); and (iii) the net economic consequences of increased expenditure for procurement of the drugs against productivity gains from increased number of patients with access to needed treatment.

This study analyses the health and health-related economic impacts over a five-year period, starting from the date on which the government use license was granted. This timeframe was decided upon to enable a fair assessment of the impact of the policy on each drug. Any health impact was assumed to occur only after the importation of the generic drugs under the licenses. Where the generic drug had not yet been imported at the time of the study, it was assumed that the drug imports would take place by January 2009. The exception was imatinib, for which implementation of the government use license has been suspended on the condition that the patent holding drug company provide the drug free of charge to patients requiring treatment, as part of the Novartis Glivec International Patient Assistance Program (GIPAP) in Thailand [14].

**Figure 1:**
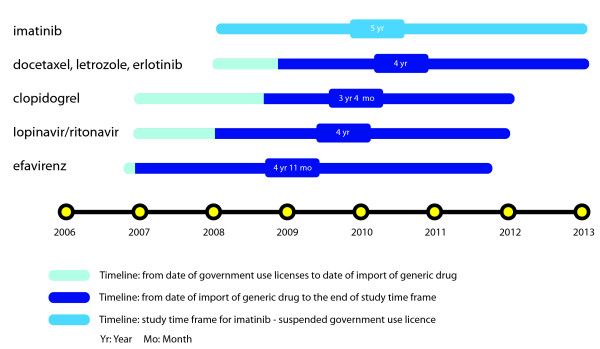
**Time frame of study in each drug**.

Since the generic drugs of EFV, LPV/r and clopidogrel were imported 1, 12, and 20 months after the issuance of government use licenses, the assessment periods for these drugs were, respectively, 4 years 11 months, 4 years, and 3 years 4 months (see Figure 1). In the case of letrozole, docetaxel and erlotinib, for which their generic equivalents had not yet been imported by the time data collection for the study had been completed (September 2008), the assessment period was 4 years (based on the assumption that imports would take place in January 2009). For imatinib, the impact assessment period was 5 years since the GIPAP agreed to provide patients under the national health insurance scheme access to the drug immediately after the grant of the government use license.

For EFV and LPV/r, the National Health Security Office (NHSO) provided data on the actual number of patients under the universal coverage scheme with access to these drugs prior to the importation of their generic equivalents. Using these data, the linear equation below calculated the number of patients who would have access to each of the drugs in the next five years, if no government use licenses had been granted.(1)

Where

Y is expected number of patients who would receive the drug

X is number of years of access to drug

u is error term

Using data on the actual numbers of patients receiving EFV and LPV/r after the importation of the generic equivalents, the same equation was also used to estimate the number of patients who will have access to the drugs in the five-year period after the grant of the government use licenses.

To determine the number of patients who had access to clopidogrel, letrozole, docetaxel and erlotinib prior to the government use licenses, the total volume of the original patented drugs imported before the grant of the government use licenses (based on data from Thai Food and Drug Administration [15]) was divided by the dosage per patient as recommended by the Ministry of Public Health (MOPH). As there was insufficient data on the number of actual patients with access to clopidogrel after the grant of the government use license, the study estimated the total number of patients who would require access to clopidogrel. This was done using data on the incidence of acute coronary syndrome in Thailand from an existing study [16,17]. The estimate was then multiplied by the total population (by age group) in 2007 [18] to estimate the total patient population in 2007, and then applied to the linear equation to estimate the future population by age group in 2007 and the total patient population for the period 2008 to 2011 [19].

For the four anti-cancer drugs there was no importation of generic equivalents at the time of the study. Therefore, data on the prevalence of lung cancer, breast cancer, GIST, and leukaemia were obtained from the Burden of Disease and Injury Project [20], estimates of incidence of such cancers were also obtained from the Thai Cancer Information Network [21], and data on the number of patients receiving imatinib were also obtained from the Novartis GIPAP [22]. These data were then applied to the linear equation to estimate the number of patients in need of each of the four cancer drugs under the government use licenses, and adjusted for dosages needed for medical treatment, in accordance to NHSO data [23] and the probability of receiving each drug, based on expert opinion from the National Cancer Institute (NCI).

Assuming an increase in the number of patients with access to the drugs, the study also sought to assess the health-related economic impact of the government use licenses from a societal perspective. That is to say, the effect of the increase in national productivity as a result of patients receiving access to drugs and the increase in health expenditure as a result of the additional drug procurement. The difference between the two figures is the net benefit to society arising from the government use licenses. Using the human capital approach, the increase in national productivity was calculated by multiplying the Gross Domestic Product (GDP) per capita with the estimated QALYs gained from the increased access to drugs as a result of the government use licenses. The study relied on a literature review of national and international publications to estimate the QALYs for the use of each drug, compared to the standard treatment used prior to the grant of the government use licenses. The total cost of each treatment option was estimated using drug prices obtained from the Drug and Medical Supply Information Center (DMSIC). Changes in the cost of treatment were determined by: (i) the difference between the prices of each drug under the government use license and its best alternative; and (ii) the difference in the cost of treating adverse effects resulting from the use of each drug under the government use license compared to its best alternatives. Non-medical costs such as infrastructure costs or patients' travel expenses to the hospital were not included as these costs should not differ between the drugs compared. All key parameters used in the analysis are presented in Tables 1 and 2 below.

**Table 1:** Input variables used in estimating health-related economic impact

Parameters	Values	References
Thai Gross Domestic Product (GDP) per capita (Baht)	135,220	[43]

Disability Adjusted Life Years (DALYs) averted from EFV-based ARVs treatment	5.69	[44]
	
DALYs averted from NVP-based ARVs treatment	5.54	

QALYs gained from LPV/r-based ARVs treatment	No-data	-
	
QALYs gained from indinavir/ritonavir (IDV/r)-based ARVs treatment	No-data	-

QALYs gained from clopidogrel plus aspirin, which is used for secondary prevention of ischemic heart disease	7.37	[45]
	
QALYs gained from aspirin alone, which is used for secondary prevention of ischemic heart disease	7.31	

QALYs gained from letrozole for treatment of breast cancer	13.14	[46]
	
QALYs gained from tamoxifen for treatment of breast cancer	12.73	

QALYs gained from docetaxel for treatment of breast cancer	0.87	[47]
	
QALYs gained from paclitaxel for treatment of breast cancer	0.66	

QALYs gained from docetaxel for treatment of lung cancer	0.41	[48]
	
QALYs gained from pemetrexed for treatment of lung cancer	0.41	

QALYs gained from erlotinib for treatment of lung cancer	0.42	[48]

QALYs gained from imatinib for treatment of Chronic Myeloid Leukemia (CML)	1.07	[49]

QALYs gained from imatinib for treatment of GIST	1.9	[50]

**Table 2:** Input variables used in estimating health care costs

Parameters	USD/yr	References
EFV-based ARVs treatment	1,922	[44]

NVP-based ARVs treatment	3,087	[44]

LPV/r-based ARVs treatment	910	DMSIC

IDV/r-based ARVs treatment	1,210	DMSIC

clopidogrel plus aspirin for secondary prevention of ischemic heart disease	19	DMSIC

aspirin alone for secondary prevention of ischemic heart disease	2	DMSIC

letrozole for treatment of breast cancer	78	DMSIC

tamoxifen for treatment of breast cancer	111	DMSIC

docetaxel for treatment of breast cancer	225	DMSIC

paclitaxel for treatment of breast cancer	1,826	DMSIC

docetaxel for treatment of lung cancer	188	DMSIC

pemetrexed for treatment of lung cancer	5,470	DMSIC

erlotinib for treatment of lung cancer	2,684	DMSIC

### The evaluation of the impact on export trade and foreign investment

The USTR withdrew the US GSP for three Thai export products; namely: 1) gold jewellery; 2) polyethylene terephthalate in primary forms; and 3) flat screen colour television sets. As reported by the media on many occasions, a Commerce Ministry official was concerned that the US GSP withdrawal for these products was due to the grant of the government use licenses and that this would have negative implications on international trade [24,25]. However, no supporting evidence has been put forward to support this claim. Note, this study does not aim to demonstrate the relationship between government use licenses and Thai exports, simply because there are many other factors at play (such as the exchange rate, price of products, specific characteristics of products or purchasing power of the buyer) [26]. Instead, this study sought to investigate the changes in national exports and in the short- and long-term foreign investment flows into Thailand following the grant of the government use licenses.

To study the effects of the government use licenses on the export values of the three products removed from US GSP scheme in July 2007, quarterly export values data between 2005 up to the third quarter of 2008 were sourced from two departments in the Ministry of Commerce, the Export Promotion and Foreign Trade Departments, and combined with data from the USTR website (http://www.ustr.gov).

Foreign investment takes place either through the classic form of foreign direct investment (FDI) or through short-term investments in the stock market. For the former, the study analysed data on applications for foreign investment licenses from 2002 to 2008 sourced from the Board of Investment of Thailand (BOI), a governmental authority which provides support and incentives to investors for medium and long-term investments. This data reflect the level of investor confidence in Thailand. The study also considered FDI applications in three industrial categories most directly related to health and research and development; namely (i) electronics and electric appliances; (ii) chemical, paper and plastic industry; and (iii) services and infrastructure industry. Particular attention was paid to the latter two categories as they are most likely to relate to the pharmaceutical industry and industries involved in the production of medical equipment and health services.

For implications on short-term investment, the study examined the changes in the Stock Exchange of Thailand (SET)'s index during the period of seven days prior to and after the grant of the government use licenses and the announcement of the withdrawal of US GSP benefits for the three products. Given that the stock market is highly sensitive to such announcements, it was assumed that it would be another good indicator of investor confidence in Thailand.

## Results

### Health-related economic impacts

The study estimated that the grant of the government use licenses would result in additional 17,959 and 3,421 patients with access to EFV and LPV/r, respectively; during the five-year study period. The estimated increase in the number of patients with access to clopidogrel was 40,947 over the five-year period. For the four cancer drugs, the estimated increases in the five-year period are as follows: 8,916 patients for letrozole; 10,813 for docetaxel, 1,846 for imatinib; and 256 for erlotinib.

The findings, in terms of QALYs gained are as follows (in order of drugs with the greatest health gains): letrozole gain of 3,656 QALYs; EFV 2,694 QALYs gained; clopidogrel 2,457 QALYs gained; imatinib: a total of 2, 435 QALYs gained (1, 384 QALYs for CML patients; 1, 051 QALYs for GIST patients); and docetaxel: 1,251 QALYs gained. There were no comparative studies available on the utility of LPV/r and erlotinib versus alternative treatments; hence, the study was not able to estimate the increase of QALYs resulting from the increased access and use of these two drugs.

The study found that health-related economic benefits to society arising from the government use licenses, as expressed in terms of the difference between national productivity and health expenditure was approximately USD132.4 million, over the five-year period. This means that the national productivity gained from the government use licenses outweighed the increase in the health budget.

In terms of the individual drugs, the greatest incremental benefit was seen in the case of EFV, as shown in Table 3. The government use license for EFV alone resulted in an incremental benefit of USD67 million. In the case of LPV/r, where data on QALYs gained were unavailable, the analysis was based on the assumption that patients receiving LPV/r will have the equivalent of QALYs gained to patients on the standard alternative treatment (i.e., IDV/r) and thus, there would be no change in net productivity. The study could therefore only assess the difference in costs, which was estimated at USD2.3 million. This was because providing access to LPV/r would result in savings for the health care budget compared to IDV/r. The incremental benefits calculated for clopidogrel was estimated at USD5.7 million. For the cancer drugs, the incremental benefits calculated for letroxole, docetaxel, and imatinib, were estimated at USD12 million, USD38.2 million, and USD7.2 million, respectively. In the case of erlotinib, because data limitations did not allow a comparison to be made to its alternative (i.e., gefitinib), the study compared the cost and productivity gained from access to erlotinib with the null scenario (i.e., 'do nothing'), for which it was found that there would be a net loss of USD0.3 million.

**Table 3:** Net and incremental benefits from the government use licenses, comparing public health expenditure prior to and after the government use licenses

Drugs	Treatment	No of patients increased access to drug (%)	Increased productivity (Million USD)	Health expenditure (Million USD)	Net Benefit (Million USD)	Incremental Benefit (Million USD) (%)
EFV	1^st ^line ARV	17,959 (21.3%)	309	97	212	**67.0** **(50.6%)**
				
NVP			301	156	145	

LPV/r	2^nd ^line ARV	3,421 (4.0%)	8.6	6.9	1.7	**2.3** **(1.7%)**
				
IDV/r			8.6	9.2	-0.6	

Clopidogrel+ASA	2^nd ^prevention of ischemic events	40,947 (48.7%)	870.6	0.9	869.7	**5.7** **(4.3%)**
				
ASA only			864.1	0.1	864.0	

Letrozole	Breast Cancer Hormone therapy	8,916 (10.6%)	343	2	341	**12.0** **(9.1%)**
				
Tamoxifen			332	3	329	

Docetaxel	Breast Cancer Chemo therapy	5,958 (7.1%)	14.6	1.3	13.3	**12.5** **(9.5%)**
				
Paclitaxel			11.1	10.3	0.8	

Docetaxel	Lung Cancer Chemo therapy	4,855 (5.8%)	5.6	0.8	4.8	**25.7** **(19.4%)**
				
Pemetrexed			5.6	26.5	-20.9	

Erlotinib	Lung Cancer Chemo therapy	256 (0.3%)	0.3	0.6	-0.3	-*
				
Gifitinib			N/A	N/A	N/A	

Imatinib	CML Chemo therapy	1,293 (1.5%)	4.1	-**	4.1	**7.2** **(5.4%)**
		
	GIST Chemo therapy	553 (0.7%)	3.1	-**	3.1	

**Total**		**84,158** **(100%)**				**132.4** **(100%)**

* Erlotinib: no data available to assess the incremental benefit

** Imatinib: no data on cost of drug as patients receive access to the drug free under GIPAP

### The impact on national economy and foreign investment

**Figure 2:**
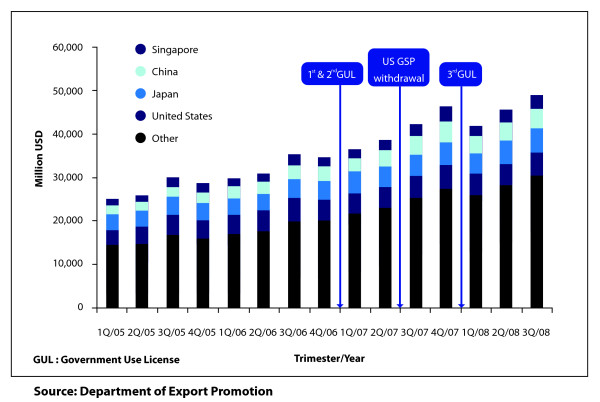
**Value of Thailand's total exports, by country, indicating timing of grant of government use licenses and withdrawal of US GSP status**.

From 1st quarter of 2005 to 3rd quarter of 2008, the total value of Thailand's exports increased steadily, particularly those to Asian countries (as seen in Figure 2). The value of exports to the US has seen minimal increase; in 2005, the figure was 16.9 billion USD, 19.6 billion USD in 2006 and 20.6 billion USD in 2007. Although still an important trading partner, the proportion of exports going to the US is clearly decreasing. As a result of the withdrawal of US GSP status, an import tariff of 6.5% was imposed on polyethylene terephthalate in primary forms, 5.5% on gold jewellery and 3.9% on flat screen colour television sets [27]. With these tariff rates, table 4 illustrates that the investment cost to the US importer for these products increased by 30.8 million USD for one year after US GSP withdrawal. This table also shows that the export values of the three US GSP withdrawal products to the US market compared one year before and after issuing the government use licenses did, in fact, decline, especially in the case of jewellery and polyethylene terephthalate. However, the export value of the same products to the rest of the world increased, thus offsetting the decrease in exports to the US market.

**Table 4:** Increased costs for US importers and changes in export value for products affected by withdrawal of GSP status (in million USD)

Product	Increased costs for US importer	Change in value of export between one year before and after US GSP withdrawal	
		
		US	Rest of the world
HS 3907.60.00 (Plastic)	0.4	-128	130

HS 7113.19.50 (Jewellery)	26	-220	723*

HS 8528.72.64 (Colour TV)	4.4	-40	-332**

**Total**	**30.8**	**-388**	**+521**

* HS 7113.19

** HS 8528

**Figure 3:**
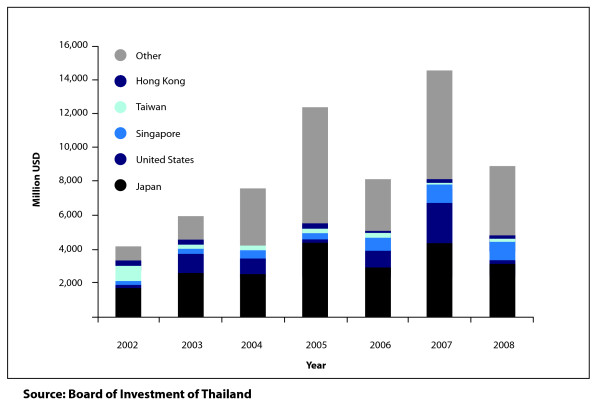
**Value of foreign direct investment interested in investing in Thailand between 2002 and 2008, by country (in million USD)**.

**Figure 4:**
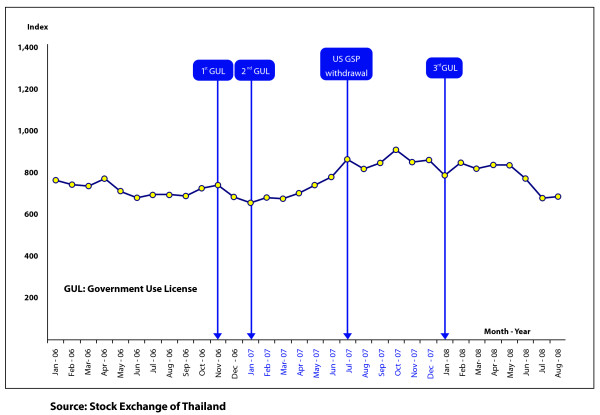
**Changes in the SET Index prior to and after the issuance of government use licenses and withdrawal of US GSP status**.

With respect to foreign investments, Thailand remains an attractive location within the international community for business investment. Figure 3 indicates that the FDI increased significantly from 2002 to 2007, rising from 4 billion USD in 2002 to 12 billion USD in 2005. FDI decreased to 8 billion USD in 2006, following the political instability in Thailand [28], but then increased again to 14 billion USD by 2007. Although, FDI flows decreased again in 2008, this is likely to be due to the global economic recession rather than the government use licenses [29]. In addition, since 2002, foreign investments in the service industry and infrastructure have increased steadily from approximately 300 million USD in 2002 to 3.5 billion USD, an 11-fold increase over a five year period. The chemicals, plastic and paper industries, and the electronics and electrical appliances industries also saw an increase in foreign investments, but less than that observed in the services industry and infrastructure. With respect to short-term investment from stock market investing professionals, both local and foreign investors, Figure 4 depicts no evidence of a link between the government use licenses or the removal of GSP status, with changes in investor confidence [30].

## Discussion

While a number of government use licenses in pharmaceuticals has been implemented in many countries, for example Indonesia, Malaysia and Ghana [31] and a number of descriptive studies have predicted that compulsory licensing could considerably increase access to drugs (see for example [3,32,33]), this impact assessment study represents, to the best of our knowledge, the first evidence-based attempt to assess the impact of the use of compulsory or government use licenses to improve access to essential drugs. The study has several strengths: the policy implications were systematically and comprehensively assessed, and data was obtained from governmental organizations including the NHSO, the MOPH's NCI and Bureau of Policy and Strategy, and the Departments of Export Promotion and Foreign Trade in the Ministry of Commerce. The availability of relevant data and the methodology used in the assessment provide a good basis for more effective monitoring of the implications of the government use licenses and for future related policy decisions in Thailand.

The study reveals a number of observations in terms of enabling the successful implementation of the government use licenses and the achievement of its maximum benefit. Table 3 illustrates that the government use licenses on EFV and docetaxel for breast cancer yield significant benefits in terms of health budget savings compared to the use of their alternative drugs while the same policy on letrozole for breast cancer has minimal health expenditure savings but generates considerable economic return from the increased productivity of those patients gaining access to the drug. This may be explained by the fact that in the case of EFV and docetaxel the differences of costs between EFV and NVP, and docetaxel and paclitaxel (see table 2); and the number of patients in need of the drugs are sizeable and this results in a significant reduction of health budget estimations. On the contrary, with the case of letrozole of which its cost differential compared to tamoxifen is not large but the clinical advantages (e.g. QALYs gained) between letrozole and tamoxifen appear to be considerable, this also creates significant economic yield from the government use licenses of the drug.

As a result, when seeking to introduce compulsory or government use licenses, a clear and transparent criterion for drug selection is recommended. This should include consideration of factors such as (i) the number of patients in need of the drug; (ii) the difference in prices between the generic drug and its alternatives; and (iii) the level of clinical advantages in terms of safety and effectiveness between the proposed generic drugs and its alternatives. Furthermore, delays in the import of clopidogrel and the cancer drugs, due primarily to the threat of prosecution by the patent holding companies [34] and political instability in Thailand, including the call for reconsideration of the government use licenses by the new government [35], are one reason for the diminished level of positive impact of the government use licenses for these drugs. It is devised that collaboration and support of key stakeholders in ensuring speedy registration, importation and distribution of the generic drug is also vital for the effective implementation of the government use license.

In contrary to the fears of significant economic losses, the study indicates that Thailand's export and FDI were not affected by the grant of the government use licenses. This may be explained by other reasons. For example, a convincing track record of growth through a very open, globally-integrated economy, privatization and widespread trade liberalisation are unique characteristics that make Thailand attractive for short- and long-term investment [36,37]. Moreover, with a strategic location at the heart of Asia, rich supply of natural resources, and a skilled and cost-effective work force, Thailand serves as a hub of exporting to Southeast Asia [38] where newly emerging markets offer great business potential.

Not many developing countries have used TRIPS flexibilities due to the discouragement of trade retaliation. For the critics, the downgrading of the country's trade status and GSP withdrawal in Thailand may discourage other countries from using this safeguard to protect their public health. However, the study's findings indicated substantial improvement in access to drugs, resulting in public health benefits for the nation, while there is no evidence of negative impact on Thai's exports and from the trade retaliation. These results can be used to inform policy makers to consider the benefit and loss of the use of TRIPS flexibilities.

This study has, however, some limitations that should be highlighted. First, the study was conducted to explore the immediate effects of the government use licenses, with particular attention paid to the health and health-related economic consequences and the impact on the national economy. It did not aim to examine other longer-term consequences, such as the potential risks of decreased levels of research and development and the impact on technology transfer. It should be noted, however, that existing studies of the effects of compulsory licensing on the incentives for pharmaceutical firms to undertake research and development and to introduce new drugs to the market are inconclusive. Some studies have indicated that drug innovation and introduction of new drugs to the market are likely to be hindered [39,40]. However, a more recent study comparing patenting rates and other measures of inventive activity before and after the grant of six compulsory licenses on drug patents in the 1980s and 1990s in the U.S. found no decline in innovation by companies affected by the compulsory licenses [41]. Another study on Canada's extensive compulsory licensing policy also concluded that it had no negative impact on pharmaceutical innovation [42].

Secondly, due to limitations of data availability at the time this study was conducted, it was assumed that all cancer patients had access to the anti-cancer drugs from the beginning of the year 2009, an assumption which might not reflect the real situation. Thus, our results are likely to overestimate the benefit of the policy on anti-cancer drugs. Thirdly, the time horizon was set at five years after the grant of the government use licenses. It is possible that the impact of the policy may be observed beyond this study timeframe. Fourthly, a number of dynamic factors influencing the effects of the government use licenses on public health and the economy may also affect the findings. For example, advancements in medical technology may lead to considerable changes in the treatment of HIV/AIDS, cardiovascular diseases and cancer. Changes in attitudes and behaviour of HIV infected patients may lead to higher rates of treatment access. Also, the drugs in question may no longer be regarded as preferred treatments for the diseases.

Finally, the findings from this study may not be applicable for assessing the impact of similar policy measures implemented in other countries. This is due to differences in health systems, disease prevalence and economic characteristics, which are strong determinants of the impact of such policy measures. However, it is suggested that the conceptual framework and methodology of the study do provide a useful basis for initiating similar efforts to monitor and assess the health and economic impacts of the use of TRIPS flexibilities for access to drugs in other developing countries.

## Conclusions

In conclusion, the study found that the government use licenses had a positive impact on national productivity due to increased access to treatment. The impact of the US GSP withdrawal did not adversely affect the overall export status. It indicated that the level of benefit gained varied according to the type of drug. This study makes recommendations for the future implementation of the policy in order to achieve its maximum benefit.

## Endnote

^i ^A compulsory license is a license granted by the government to allow someone else to produce the patented good or replicate the process without the consent of the patent owner. When a government itself uses, or authorizes a third party to use, a patented invention for government purposes, without the permission of the patent holder, this is called a government use.

^ii ^The system that extends the preferential access to the markets to certain products originating in designated developing countries.

## Competing interests

The authors declare that they have no competing interests.

## Authors' contributions

YT and ST have made contributions to conception and design. IY, AM, KC have acquired, analyzed and interpreted data. All the authors have been involved in drafting and revising the manuscript and have read and approved the final manuscript.
